# AIMedGraph: a comprehensive multi-relational knowledge graph for precision medicine

**DOI:** 10.1093/database/baad006

**Published:** 2023-02-28

**Authors:** Xueping Quan, Weijing Cai, Chenghang Xi, Chunxiao Wang, Linghua Yan

**Affiliations:** Department of Artificial Intelligence, Shanghai Tongshu Biotechnology Research Institute, No26 and 28, 377 Lane of Shanlian Road, Baoshan District, Shanghai 200444, China; Department of Innovative Technology, Shanghai Tongshu Biotechnology Research Institute, No26 and 28, 377 Lane of Shanlian Road, Baoshan District, Shanghai 200444, China; Department of Artificial Intelligence, Shanghai Tongshu Biotechnology Research Institute, No26 and 28, 377 Lane of Shanlian Road, Baoshan District, Shanghai 200444, China; Department of Innovative Technology, Shanghai Tongshu Biotechnology Research Institute, No26 and 28, 377 Lane of Shanlian Road, Baoshan District, Shanghai 200444, China; Department of Innovative Technology, Shanghai Tongshu Biotechnology Research Institute, No26 and 28, 377 Lane of Shanlian Road, Baoshan District, Shanghai 200444, China

## Abstract

The development of high-throughput molecular testing techniques has enabled the large-scale exploration of the underlying molecular causes of diseases and the development of targeted treatment for specific genetic alterations. However, knowledge to interpret the impact of genetic variants on disease or treatment is distributed in different databases, scientific literature studies and clinical guidelines. AIMedGraph was designed to comprehensively collect and interrogate standardized information about genes, genetic alterations and their therapeutic and diagnostic relevance and build a multi-relational, evidence-based knowledge graph. Graph database Neo4j was used to represent precision medicine knowledge as nodes and edges in AIMedGraph. Entities in the current release include 30 340 diseases/phenotypes, 26 140 genes, 187 541 genetic variants, 2821 drugs, 15 125 clinical trials and 797 911 supporting literature studies. Edges in this release cover 621 731 drug interactions, 9279 drug susceptibility impacts, 6330 pharmacogenomics effects, 30 339 variant pathogenicity and 1485 drug adverse reactions. The knowledge graph technique enables hidden knowledge inference and provides insight into potential disease or drug molecular mechanisms.

**Database URL**: http://aimedgraph.tongshugene.net:8201

**Key Points**

- AIMedGraph collects and interrogates standardized information about genes, genetic alterations and their therapeutic and diagnostic relevance and builds a multi-relational, evidence-based knowledge graph.
- The knowledge graph enables hidden knowledge inference and provides insight into potential disease or drug molecular mechanisms.
- The Web Application Interface version of AIMedGraph is freely accessible to users with different biomedical backgrounds.

## Introduction

Comprehensive molecular profiling of various tumors leads to the concept of ‘personalized’ or ‘precision’ medicine (1). Precision medicine has played emerging roles in guiding clinical decisions, particularly in disease diagnosis and drug therapy (2–12). Although providing inadequate information, multi-omics data are essential and applicable for the diagnosis and treatment of patients, especially with solid malignant metastatic tumors (1, 7, 9, 13). In the past decade, with the fast-developing next-generation sequencing techniques, biomedical scientists, genetic epidemiologists and pharmaceutical scientists are able to investigate the impact of genetic differences between individuals on their susceptibilities to diseases/drugs on large sample scales, generate evidence about the associations between genetic and phenotypic variations on the population level, reveal the genetic mechanisms of disease development and treatment action and develop molecular companion diagnosis kits and targeted therapies or interpret individual’s genetic variation profile.

However, information about the impact of omics variation from DNAs, RNAs, proteins and metabolites on clinical diagnosis/treatments and related clinical actionable information are distributed in different types of databases, publications and guidelines. Several databases have been developed to curate data from both omics and clinical sides. Comprehensive databases like the Online Mendelian Inheritance in Man (14) give brief, unstructured descriptions about genetic disorders and their associated genes. Orphanet (15) and Genetic and Rare Diseases (16) collect and classify rare diseases with involved genes and provide an inventory of orphan drugs. The Human Gene Mutation Database (17) manually curates information on germline mutations associated with inherited diseases, covering DNA-level mutations including missense mutations, nonsense mutations and splice-site mutations. The Catalogue Of Somatic Mutations In Cancer (COSMIC) (18) collects multiple types of somatic mutations detected in human cancers. Clinvar (19), as an open database, collects all disease-associated genetic mutations, providing phenotype information, pathogenicity evaluations of these mutations and functional annotation based on Gene Ontology (20). There are also some oncology-specific databases like Clinical Interpretations of Variants in Cancer (CIViC) (21), knowledge graph for hepatocellular carcinoma (KGHC) (22), OncoKB (23), OncoTree (24) and PharmGKB (25), which collect the impact information of genetic variants on targeted drug response, cancer diagnosis or prognosis. These databases, whether comprehensive or field-specific, adopt relational database techniques and store variants, diseases and drugs as rows and columns, and their relationships are given.

It is well documented that precision medicine knowledge is highly enriched by studies on multi-omics data, and the key components are the relationships between all the different omics entities (7, 26–34), e.g. variants, diseases and drugs, each at the scale of thousands to hundreds of thousands. Knowledge graph techniques can help to construct a comprehensive view of these entities and their relationships through a process called semantic enrichment. It allows question answering and search systems to retrieve and reuse comprehensive answers to given queries. The graph-based architecture to represent relations also supports the creation of new knowledge, establishing connections between data points that may not have been realized before. DisGeNET (35) has managed to build a knowledge graph on gene–disease or variant–disease associations inferred from studies like genome-wide association studies. However, there is currently still no knowledge graph providing evidence-based variant–drug relations, which is the most important information needed for precision medicine (1, 2, 36–38).

**Figure 1.**
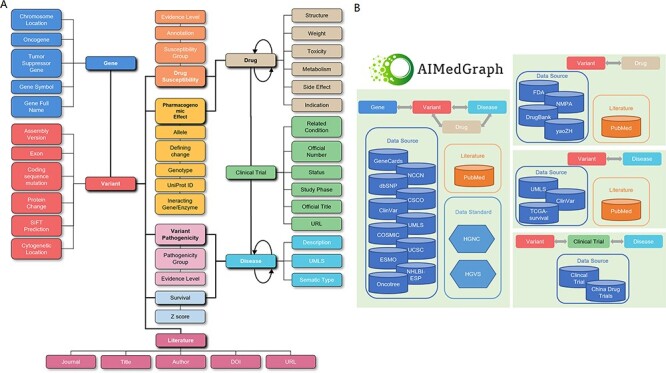
The AIMedGraph knowledge graph. (A) Simplified AIMedGraph architectures; (B) AIMedGraph data sources.

This article describes the methodology of AIMedGraph, which represent and integrate precision medicine knowledge into multiple relations. The AIMedGraph knowledge graph curated detailed information about diseases, drugs, genes, genetic variants and the impact of genetic variations on disease development and drug treatment from multiple data resources (Figure 1) in an evidence-based medicine approach. It laid the basis of a self-developed querying and answer system. Based on a multi-relational knowledge graph, users with various biological and medical backgrounds can visualize variant–drug relationships and get inferred information about drug development.

## Materials and methods

### Precision medicine knowledge deconstruction and representation

The first step to developing a knowledge graph is to define the entities, the classification and direction of relationships between entities and the attributes describing the entities for the communication and integration between different data resources. In general, we need to deconstruct precision medicine knowledge and develop data models to represent knowledge in a way computers could understand and process. The main entities in precision medicine knowledge are defined to be genes, variants, diseases, drugs, clinical trials and supporting evidence levels. Relation classes include the relations between diseases, such as subtypes, stages and complications of diseases, relations between genes and diseases, relations between genetic variants and diseases, the pathogenicity of variants for a disease, the indications of drugs, interactions between drugs, the impact of variants/genes on drug therapy, the disease, drug and gene/variant a clinical trial study on and the literature studies supporting entity or relationship (Figure 1A).

### Clinical entity data model and relationships

#### Drug data model

The data model of drugs collects their common database ID, type, name, synonym, Chinese name, trade name, drug target, indication, instruction, chemical structure, molecule weight, chemical formula, pharmacology information including mechanism of action, metabolism, toxicity, adverse effects and the drug–drug interaction, consisted of interacting drugs and effects (Figure 1; Supplementary Table S1). Drugs are classified into being either small molecules or biotech drugs.

#### Disease classification

**Figure 2.**
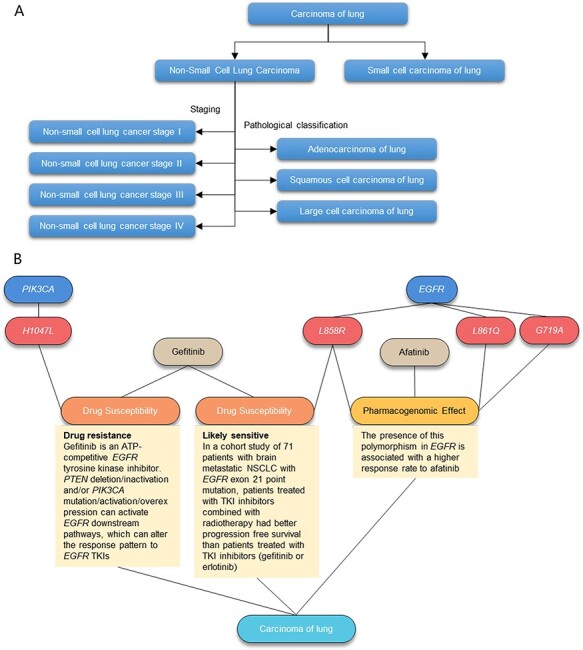
Illustration of the AIMedGraph relation graph. (A) Example of the disease classification tree. (B) Example of the relation graph between genes, variants, drugs and diseases.

Diseases in AIMedGraph refer to a wide range of medical terms including pathological diagnosis names, disease stages, symptoms, molecular typing and normal traits. The Unified Medical Language System (UMLS) was adopted to standardize medical terms and codes and builds the semantic relationship between diseases. These medical terms are further organized into a classification tree by expert clinicians with reference to pathology classification, disease stage, metastasis, reoccurrence and molecular typing (Figure 2A) and oncology diseases were classified with reference to OncoTree (24). For instance, there are Stages I, II, III and IV of non-small cell lung cancer, as well as pathological squamous cell, adenocarcinoma and large cell of lung cancer (Figure 2A). A brief description directly extracted from the UMLS was also provided for each disease entity.

#### Clinical trial data model

The data model for clinical trials in AIMedGraph collects information registered on the China drug trial site (www.chinadrugtrial.cn) and the US clinical trial site (www.clinicaltrial.gov). Attributes defined in this data model include the official title, official number, indication, recruiting status and study phase (Figure 1; Supplementary Table S1).

### Omics entity data model and relationships

Genes collected in AIMedGraph are mainly protein-coding genes. Information about genes and their variants were extracted from the National Center for Biotechnology Information’ public databases Entrez (http://www.ncbi.nlm.nih.gov/Entrez/), Ensembl (39), 1000 genomes (40) and the Single-Nucleotide Polymorphism database (41). Summary information about genes includes their gene names, synonyms, brief descriptions and related clinical trials. Basic information about genes collects more features, including being oncogene or not, being tumor suppressor gene or not, external database IDs, human genome (HG) position on chromosomes and reference genome assembly version (Supplementary Table S1).

Attributes collected for variants include the gene variant location, CoDing Sequence (CDS) change, amino acid change, transcript ID, HG position, exon located on, variant type, amino acid change type and the Sorting Intolerant from Tolerant prediction. Different types of variations differ on some features as structural variants like fusion and copy number variation do not have CDS change and amino acid change information. Supplementary Table S1 lists the features collected for variants with a curated data model and collected information in AIMedGraph. The nomenclature of molecules and variations follows international standards set by the Human Genome Organisation Gene Nomenclature Committee and the HG Variation Society (HGVS) and was normalized and corrected by self-developed script following the international standard HGVS to use the most 3ʹ-end position of the transcript when aligning variant sequence to the reference genome. Pharmacogenetic haplotype markers, which are groups of variants, follow the star allele nomenclature (42).

### Clinical and omics association

The development of disease, or efficacy of treatment, is affected by variations in different genes. The impacts of omics variants on clinical information are further divided into three categories: disease pathogenicity, drug susceptibility and pharmacogenomics.

Variant–disease pathogenicity relations were mostly extracted from ClinVar. The variant that was pathogenic or benign with supporting evidence on the top three ClinVar evidence levels (practice guideline, review by expert panel and criteria provided by multiple submitters with no conflicts) was collected. Criteria used follow the American College of Medical Genetics and Genomics guideline and the Association for Molecular Pathology guideline (43). According to its host or major neighbor gene of a variant, gene–disease relationships, together with a brief description and a score of the evidence, were directly extracted from DisGeNET (35).

Drug susceptibility, that is, drug response association, mainly collects the impact information of molecular variations on treatment, including targeted therapy, immune therapy, hormone therapy and chemotherapy. Drug response information was retrieved from the Food and Drug Administration (FDA) of the United States, the National Medical Products Administration of China, clinical guidelines like the National Comprehensive Cancer Network, the European Society for Medical Oncology and the Chinese Society of Clinical Oncology and literature studies in PubMed and manually reviewed by expert genetic consultants (Figure 1, Supplementary Table S1). The response of variants to drugs are grouped into four categories: sensitive, likely sensitive, likely resistant and resistant, with clinical annotations that are curated descriptions about supporting study design, sample size, sampling population and related numerical indexes overall response rate, overall survival, progression-free survival, etc.

Pharmacogenomics information was extracted from DrugBank and covered interacting gene/enzyme, allele name, gene name, genotype, nucleic acid change, evidence type, UniProt ID, description and reference, as shown in Figure 1 and Supplementary Table S1.

### Relationship graph

Graph database Neo4j provides the technical foundation to store, manage and visualize all the attributes and relationships described earlier (Figure 2B). Relationships curated in AIMedGraph include the disease classification, drug–drug interaction, gene–variant ownership (*PIK3CA-H1047L; EGFR-L858R/L861Q/G719A*; Figure 2B), variant–disease pathogenicity, variant–drug–disease drug susceptibility (Gefitinib resistant to lung cancer with the *PIK3CA H1047* variant, but likely sensitive to patients with the *EGFR L858R* variant; Figure 2B), variant–drug–disease pharmacogenomic impact (Afatinib has both sensitive and pharmacogenomic effects in case of the *EGFR L858R*-positive variant in lung cancer; Figure 2B), clinical trial–drug–indication recruitment condition and supporting reference to all these relations.

### AIMedGraph Application Programming Interface

AIMedGraph is available as a web application programming interface on http://aimedgraph.tongshugene.net:8201 to query, analyze and visualize the content of AIMedGraph. Information will be extracted via keywords for specific genes, variants, diseases, drugs or literature studies. The search results are organized into relationship graphs and tables in six separate modules to provide different views of the information (Supplementary Table S1). The front page also provides entity statistics of the currently released version.

AIMedGraph Application Programming Interface (API) is an integration of multiple modern techniques, including graph database Neo4j for the relation storage, ElasticSearch + mysql for the data storage, Sprint Boot and Redis for the backend service, Vue and ElementUI for the front-end framework and NeoVis for the relation graph, allowing a comprehensive set of functions of the API to query, visualize and mining the attributes and relations of all the key components.

### Computing algorithm of relation prediction

The entities integrated into AIMedGraph are connected by evidence-based relations and form a comprehensive gene–variant–disease–drug–trial–reference knowledge network. It provides a solid basis for novel relation prediction.

The reliability of the predicted link is measured by the Adamic–Adar algorithm based on the shared neighbors between two nodes. It is computed using the following formula:

$$A\left( {x,y} \right) = \sum\limits_{u \in N(x) \cap N(y)} {\frac{1}{{\log \left| {N(u)} \right|}}} $$

where *N*(*u*) is the set of nodes adjacent to *u*. A value of zero for *A*(*x*, *y*) indicates that nodes *x* and *y* are not close to each other, while a higher value indicates closer relation.

The average reliability value for the 10 519 predicted drug–indication relations is 1.629, while the average reliability value for the known 680 drug–indication relations is 9.997. Targeted therapies like gefitinib, Afatinib or Erlotinib have very high values on their relations with non-small cell lung cancer (NSCLC), being 45.578, 34.010 or 35.857, respectively. Similarly, relations like imatinib–gastrointestinal stromal tumors and pembrolizumab–melanoma all have very high values (42.533 and 55.503, respectively). This proved the reliability of the Adamic–Adar algorithm for link prediction. In summary, as a comprehensive knowledge graph, AIMedGraph enables the efficient analysis and interpretation of genetic profiles.

## Results

### Statistics of AIMedGraph

To date, there are 187 541 variants curated in AIMedGraph where there are pieces of evidence for their significant associations with disease or treatment. As shown in Table 1, about 2821 drugs and 30 340 diseases/traits/phenotypes are associated with these variants/genes, with 33 784 associations on variant pathogenicity, 9279 on drug susceptibility related to 758 targeted therapies and 598 diseases and 6330 on pharmacogenomics effect. Adverse effect information was curated for 1485 drugs as well. Enrollment information for 15 125 clinical trials related to these drugs or diseases was also curated into AIMedGraph for patients to get involved. Compared with the DisGeNET platform, AIMedGraph contains more diseases and genes a little bit. More importantly, only drug–disease relations and variant–drug relations can be searched and presented in AIMedGraph, not in DisGeNET, such as drug susceptibility impacts, pharmacogenomics effects and drug interactions. Because OncoKB is a specific precision oncology knowledge base, there are a total of 113 drugs available for 133 cancer types, far <348 drugs for various cancer types in AIMedGraph.

**Table 1.** Metrics comparison between AIMedGraph, DisGeNET and OncoKB

	AIMedGraph	DisGeNET	OncoKB
Relationship	242 901	1 134 942^a^ + 369 554^b^	NA
Disease	30 340	30 170	133
Gene	26 140	21 671	688
Variant	187 541	194 515	5753
Drug	2821	NA	113
Drug interaction	621 731	NA	NA
Drug susceptibility	9279	NA	NA
Pharmacogenomics effect	6330	NA	NA
Variant pathogenicity	33 784	NA	NA
Drug adverse reactions	1485	NA	NA
Clinical trails	15 125	NA	NA
Relation visualization	Multi-relational	NA	NA

NA: not applicable.

a Gene–disease associations.

b Variant–disease associations.

The 187 541 variants are diverse in types, including single-nucleotide variant (SNV), insertion, deletion, indel, complex, inversion, translocation, duplication, copy number gain, copy number loss, microsatellite, variation that is a large chromosome-level change, pharmacogenetic haplotype, haplotype single variant and diplotype that follows the star allele nomenclature (Table 2). There are 187 172 variants that are related to/locate on 6548 genes. There are 15 367 genes coming from DisGeNET that are directly associated with diseases without specific variant connections. Among the total 26 140 genes integrated into AIMedGraph, 262 were annotated to be an oncogene, and 273 to be tumor suppressor genes.

**Table 2.** AIMedGraph variant metric

Variant type	Number
SNV	166 883
Insertion	811
Deletion	9966
Indel	944
Duplication	4254
Copy number gain	30
Copy number loss	30
Complex	1
Microsatellite	2867
Inversion	66
Translocation	151
Variation (a chromosome-level change)	33
Haplotype, single variant	21
Haplotype	23
Diplotype	595

### Distribution of drug susceptibility in AIMedGraph

**Figure 3.**
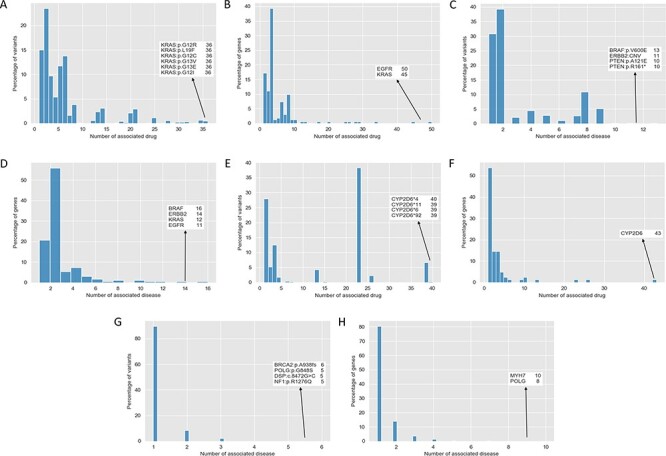
Distribution of drug susceptibility, pharmacogenomics effect and variant pathogenicity. (A) Distribution of the number of associated drugs per variant with drug susceptibility impacts. (B) Distribution of the number of associated drugs per gene with drug susceptibility impacts. (C) Distribution of the number of associated diseases per variant with drug susceptibility impacts. (D) Distribution of the number of associated diseases per gene with drug susceptibility impacts. (E) Distribution of the number of associated drugs per variant with pharmacogenomics effects. (F) Distribution of the number of associated drugs per variant with pharmacogenomics effects. (G) Distribution of the number of associated diseases per variant with pathogenicity. (H) Distribution of the number of associated diseases per gene with pathogenicity.

The majority of variants with drug susceptibility impacts are associated with less than seven drugs (15.02% with one, 23.45% with two, 9.73% with three, 5.36% with four, 11.72% with five and 13.79% with six drugs). Similarly, 67.48% of genes are associated with less than four drugs (17.18% with one, 11.04% with two and 39.26% with three drugs) (Figure 3A and B). The susceptibilities of 36 drugs are affected by six *KRAS* variants, *G12R, L19F*, *G12C*, *G13V*, *G13E* and *G12I*, respectively. On the gene level, *EGFR* and *KRAS* all accumulate drug susceptibility variants, with *EGFR* associated with 50 drugs and *KRAS* with 45 drugs (Figure 3A and B). The indications associated with these drug susceptibility variants are less evenly distributed with 30.89% of variants associated with only one indication and 39.23% with two indications (Figure 3C). Some variants, including *BRAF V600E*, the copy number variation of *ERBB2*, *PTEN A121E* and R161*, are associated with >10 cancer indications and form hubs of the network. Similarly, most of the genes these variants locate on are associated with one (20.86%) or two (55.83%) indications, with exceptional *BRAF*, *ERBB2*, *KRAS* and *EGFR* associated with >10 types of cancer indications (Figure 3D).

### Distribution of pharmacogenomics in AIMedGraph

As illustrated in Figure 3E and F, the distributions of pharmacogenomics effects are less smooth and have multiple peaks. The top peak, 38.26% variants, is associated with 23 drugs. And the second peak, 27.964% variants, is associated with only one drug. Four haplotypes of the most polymorphic gene *CYP2D6*, which metabolizes ∼20% of drugs (1250 *CYP2D*6 variant-related drugs/total variant-related drugs 6055 in AIMedGraph), CYP2D6*4, CYP2D6*11, CYP2D6*6 and CYP2D6*92, are associated with 40, 39, 39 and 39 drugs, respectively. Additionally, the *CYPD26* gene is associated with 43 drugs, while 53.7% of genes are associated with 1 drug only.

### Variant pathogenicity in AIMedGraph

Another important type of relations in AIMedGraph is the pathogenicity of variant to disease. In Figure 3Gand H, the majority (89.52%) of variants are pathogenic or benign to one type of disease with a high level of supporting evidence (26 808 single relations over 29 946 total relations of variant–disease in AIMedGraph). A few variants like *BRCA2 A938fs* (43), *POLG G848S* (44, 45) and *NF1 R1276Q* (43, 46) have been proven to be pathogenic and leading to multiple diseases, while *DSP 8472G>C* has been proven to be benign for five conditions by multiple researchers (43, 47).

### Friendly user query–answer and knowledge graph interpretation via AIMedGraph

AIMedGraph has a friendly user interface to support making a query with single or multiple keywords, like gene, variant, drug, disease, clinical trial and literature. Simple searching for the shared variants of drug susceptibility and variant pathogenicity relations yields 2153 potential drug–disease candidates. For example, Breast carcinoma patients with variant *PIK3CA p.Glu453Lys* are sensitive to the FDA-approved therapy Alpelisib + Fulvestrant based on the result of a Phase III clinical trial SOLAR-1 (NCT02437318). This variant has been labeled to be pathogenic to megalencephaly cutis marmorata telangiectatica congenita. So Alpelisib + Fulvestrant may work as a candidate for megalencephaly cutis marmorata telangiectatica congenita drug development. Another example of inferred relationship is that colorectal carcinoma patients with the *NF1 p. Ile679fs* variant are likely resistant to the drug Cetuximab and may not benefit from this drug based on a clinical study with 33 Chinese metastatic colorectal cancer patients (13). However, the drug selumetinib has been approved by the FDA in 2020 to treat neurofibroma patients for >2 years. This approval is based on a Phase II clinical trial and showed that patients with the *NF1 p. Ile679fs* variant are sensitive to selumetinib (NCT04924608). It is likely that CRC patients with *NF1 p. Ile679fs* are sensitive to selumetinib as well.

**Figure 4.**
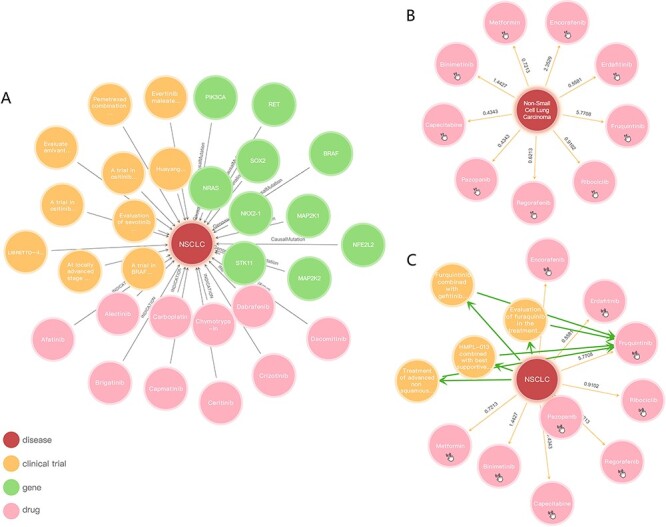
Knowledge graph presentation of NSCLC: (A) NSCLC relationships between genes, drugs and clinical trials, respectively; (B) inferred relationships of NSCLC; (C) reasoning between NSCLC and Fruquintinib.

It is well-known that NSCLC is the most popular and lethal tumor disease globally. Using NSCLC as a keyword to search answer in AIMedGraph, a knowledge graph of NSCLC is instantly presented just like it shown in Figure 4A. Around the disease NSCLC, 10 genes, 10 drugs and 10 clinical trials are linked with different specific relationships, including genomic alteration, causal mutation, indication and trial. If one is interested in NSCLC’s reasoning relationship, the inferred information would be displayed by clicking the button of AIReasoning. A new graph displayed in Figure 4B states that the new nine drugs are associated with NSCLC. Because the digital number represents the stronger relationships between two metrics, we selected Fruquintinib as an example with a value of 5.77, higher than the average reliability value of 1.63. By clicking Fruquintinib, four new relations of clinical trials (NCT02590965, NCT02976116, NCT02691299 and NCT03684967) between NSCLC and Fruquintinib could be established, respectively, in Figure 4C. Thus, we can assume that Fruquintinib may be considered as an interval drug to treat NSCLC.

## Discussion

With the aim to provide easy exploration and comprehensive visualization of the treatment and disease information related to a patient’s specific genetic profile, the development of AIMedGraph is focused on the semantic integration and active visualization of multidimensional information from multiple resources. The databases integrated by AIMedGraph include omics databases, disease databases, drug databases and structured information extracted from unstructured literature studies and guidelines. Reasonably, the relationships among different omics data could be useful and helpful in precision medicine. Although the present integration of omics data is inadequate, it clearly indicates that the multi-relational knowledge would be essential to understand precision medicine more accurately (2, 28, 32, 34, 48, 49). AIMedGraph seems to be tailored for different users who are interested in personalized management of disease.

Quality control of supporting evidence and accurate presentation of evidence in a structured way are other important factors. All the information retrieved from authorized public databases must be brought into line with international standards, and the detailed items should be structured. The precision medicine is characterized by molecular pathological diagnosis and targeted therapy, underlining genome sequencing data of diseases, drug susceptibility, pharmacogenomics and clinical trials. Properly analyzing and accurately interpreting are critical essential components, allowing personalized diagnosis and treatment according to the information from the individual patient’s unique genetic profile and specific environmental factors (50). Via quality control, individual variability in genes, environment and lifestyle factors, the standard of care in oncology and targeted drug therapies could be accurately interpreted in a structured manner. It is undoubtedly that overinterpretation or misinterpretation would all lead to the treatment of patients with ineffective but expensive therapies, negatively affecting not only patient lives but also the health care budget (1). Therefore, quality control is necessary for proper interpretation.

AIMedGraph is convenient for users to find the information they wanted whether their initial clue is a gene, a variant, a drug or a disease that they could remember. The graph-based infrastructure connecting different entities with labels on the relationships between these entities can enable the extraction of information along the graph path in two or three steps. For example, if a query is a gene name, users could get disease information in just one step from the gene–disease relationship, or two steps from the gene–variant ownership and variant–disease pathogenicity, or drug information in two steps from gene–variant ownership and variant–drug susceptibility or variant–drug pharmacogenomics effect; further clinical trial information could be obtained via drug–clinical trial relations. Its convenience might simplify patients’ education via knowledge graph (Figure 4) because patients would not have to keep too many professional terms in mind. In the real world, an improvement in patients’ awareness of molecular testing would play a positive role in medical care, clinical outcome and life quality (51–54).

Actually, there are a couple of databases or platforms involved in precision medicine, such as Pharmacogenomics (25, 38), DisGeNET (35), CIViC (21, 37), COSMIC (18), HGen (55), the Immuno-Oncology Biological Research (33), KGHC (22), MedGen (56), OncoTree (24) and OncoKB (23, 38). Based on gene polymorphisms, Pharmacogenomics provides medication selection with different dosages to minimize potential drug toxicities in the treatment of relative diseases, including cancers, depression disorder and hypertension (25, 38). DisGeNET is an interoperable resource focusing on gene–disease and variant–disease associations (35). OncoKB is an expert-guided precision oncology knowledge base that can interpret how somatic molecular alterations predict drug response for various cancer types (23, 38). In comparison to OncoKB, the CiViC knowledge database has a highly similar goal and extracts the information data from the identical data sources. But CIViC has the largest number of unique drugs and the largest number of unique gene-drug associations (37).

Compared with the above well-developed and commonly used databases or platforms, AIMedGraph has several merits. First, AIMedGraph adopted the knowledge graph technique that provides an effect to store and extract the multimodel relations between all the different omics and clinical factors. Through a relation graph, all information related to a query point could be displayed at the same time for comprehensive visualization. Information mining and inferred relationships from AIMedGraph could provide insight into putative mechanisms and boost clinical practice and research on drug development. In regarding this point, Pharmacogenomics, DisGeNET, CIViC and OncoKB have no such function. Second, not only the information on the associations betweeen gene/variant and diseases are integrated into AIMedGraph, similar to DisGeNET, but also clinical actionable information, including the impact of variant on drug susceptibility, drug effect/dosage and adverse effect, and variant pathogenicity are integrated into AIMedGraph as well, together with supporting annotation and evidence level. Just like drug repurposing studies (49, 57–63), AIMedGraph could prioritize drug repurposing medications through our AI-Reasoning assistive tool, particularly in antitumor drugs. In general, AIMedGraph integrates multidimensional, evidence-based knowledge to interpret genetic variants for efficient clinical and research recommendations.

## Supplementary Material

baad006_Supp

## Data Availability

AIMedGraph is freely accessible at http://aimedgraph.tongshugene.net:8201.
