# Supplementary material for: AIMedGraph: a comprehensive multi-relational knowledge graph for precision medicine
Source: Database (Oxford). 2023 Feb 28;2023:baad006. doi: 10.1093/database/baad006 (PMC9976745; doi:10.1093/database/baad006)
Supplement: baad006_Supp [file baad006_supp.zip › suppl_data/Supplementary Table 1.pdf]

Supplementary Table 1. Information recorded for disease, drug, clinical trial, gene, variant, supporting literature, and each type of relationship.

| Entity | Category | Attribute | Entity | Category | Attribute |
|---|---|---|---|---|---|
| Gene | Summary | Gene name | Drug | Summary | Drug Name |
| | | Alias names | | | Type |
| | | Gene Description | | | Drug Synonyms |
| | | Clinical Trails | | | Disease Name |
| | Basic Information | Gene name | | Basic Information | Drug Name |
| | | Synonyms | | | Drug Synonyms |
| | | Gene Description | | | Trade name |
| | | HG Position (Assembly Position) | | | Target |
| | | Is Oncogene | | | Indication |
| | | Is Tumor Suppressor Gene | | | FDA Approved Indication |
| | | External IDs | | | NMPA Approved Indication |
| | Related Clinical Trials | Official Title | | | Drug Type |
| | | Offical Number | | | Drug instruction |
| | | Study Phase | | | Chemical structure |
| | | Disease | | | Weight |
| Variant | Summary | Gene name | | | Chemical Formula |
| | | HGVSc | | Pharmacology | Mechanism of Action |
| | | Protein.Change | | | Metabolism |
| | | Gene Location | | | Toxicity |
| | | SIFT Prediction | | | Adverse Effects |
| | | Associated cancer types | | Drug Interaction | Interacting Drugs |
| | | Associated drugs | | | Interacting Effect |
| | | Clinical evidence | | Pharmacogenomics | Interacting Gene/Enzyme |
| | | Reference | | | Allele Name |
| | Basic Information | Genome Sequence Change | | | Gene Name |
| | | Coding Sequence Change | | | Genotype |
| | | Amino Acid Change | | | Nucleic Acid Change |
| | | Gene Location | | | Type |
| | | Affected Exon Number | | | UniProt ID |
| | | Variant.Type | | | Description |
| | | SIFT Prediction | | | References |
| | Clinical Impact | Clinical Impact Category | Disease | Summary | Associated Genes |
| | | Clincal effect | | | Clinical Trails |
| | | Evidence level | | | Associated Drugs |
| | | Associated disease | | | Disease Description |
| | | Associated drugs | | | Reference |
| | | Clinical annotation | | Basic Information | Disease Description |
| | | Reference | | | disease name |
| Clinical Trial | Basic Information | Official Number | | | Gene name |
| | | Related Conditions | | | Alterations |

| | | | | | |
|---|---|---|---|---|---|
| | | Recruiting Status | Clinical Interpretation | Basic Information | Efficacy |
| | | Study Phase | | | Level of Evidence |
| Reference | Basic Information | DOI | | | Clinical Annotation |
| | | Author | | | |
| | | Journal | | | |
